# Proteomic differences in seminal fluid of social insects whose sperm differ in heat tolerance

**DOI:** 10.1098/rsos.231389

**Published:** 2023-11-08

**Authors:** Baptiste Martinet, Kimberly Przybyla, Corentin Decroo, Ruddy Wattiez, Serge Aron

**Affiliations:** ^1^ Evolutionary Biology & Ecology, Université Libre de Bruxelles, Avenue Paul Héger - CP 160/12, 1000 Bruxelles, Belgium; ^2^ Department of Zoology, Université de Mons, 7000 Mons, Belgium; ^3^ Department of Proteomics and Microbiology, Université de Mons, 7000 Mons, Belgium

**Keywords:** bumblebees, global decline, thermal tolerance, heat exposure, seminal fluid, proteomics

## Abstract

In the coming years, climate change is likely to increase the frequency and intensity of heatwaves. In many organisms, heat stress provokes physiological perturbations and can lead to decreased male fertility. Bumblebees are endo-heterothermic but display interspecific differences in thermotolerance that could have conservation implications. For the species of concern *Bombus magnus*, exposure to high temperatures can severely reduce sperm quality and, consequently, reproductive success. Such is not the case for *B. terrestris*, a ubiquitous species. To decipher the mechanisms at play, we characterized the seminal fluid proteomes of the two species. We quantified 1121 proteins, of which 522 were differentially expressed between *B. terrestris* and *B. magnus*. Several proteins with protective functions, such as proteases, antioxidant proteins and various heat-shock proteins, were present at higher levels in *B. terrestris* than in *B. magnus* under both control and heat-stress conditions*.* The same was true for proteins involved in cellular homeostasis, immunity, lipid/sugar metabolism and thermotolerance. Furthermore, proteins involved in the capture and elimination of reactive oxygen species also occurred at much high levels in *B. terrestris*. Overall, these results clearly indicate differences in the seminal proteome of the more thermotolerant *B. terrestris* versus *B. magnus*. The differences may contribute to explaining interspecific differences in sperm survival.

## 1. Introduction

Worldwide, biodiversity is in steep decline. The chief driver is anthropogenic climate change [1], which impacts ecosystems by modifying the geographical ranges of species [2,3] and by causing local extinctions or biological invasions [4]. Models predict the planet will experience more frequent extreme climatic events, including heatwaves [5,6]. Heatwaves can dramatically affect biodiversity because (i) their swiftness may not allow organisms to adapt [7] and (ii) their brief, stochastic, and unusually extreme thermal conditions negatively impact biological functions [8]. Over the last decade, increasing attention has been paid to the consequences of heat exposure for biodiversity in terrestrial, marine and freshwater ecosystems [9,10]. Yet, knowledge remains scarce on the proximate drivers behind population declines in diverse taxa such as insects [11].

As heterotherms, insects are particularly sensitive to extreme temperature variation, which can result in major physiological perturbations (e.g. decreased fertility) and, in some cases, death [12,13]. At present, little research has been dedicated to understanding the consequences of heatwaves on reproduction in insects. High temperatures can negatively affect male courtship behaviour in solitary bees in the genus *Osmia* and in bumblebees [14,15]. Moreover, exposure to a heat shock can decrease sperm quality in flies (*Drosophila*), beetles (*Tribolium castaneum*), parasitic wasps (*Anisopteromalus calandrae*), domesticated bees (*Apis mellifera*) and bumblebees (*Bombus jonellus*, *B. magnus*) [15–19].

Sperm vulnerability to heat presents a particular risk in social hymenopterans (i.e. ants, bees and wasps). This large group of insects comprises more than 18 000 living species, including many that provide key ecosystem services, such as pollinating plants, controlling other insects and dispersing seeds [20]. All male hymenopterans are sperm limited—they start life with the only sperm reserves they will ever have. Typically, sperm production occurs during the nymphal/pupal stage and ends shortly after emergence, as the testes degenerate [21]. The resulting sperm are then stored in the accessory testes until mating, which happens in early adulthood for both males and females. Males die shortly after copulation, from exhaustion and/or predation, but persist posthumously in their spermatozoa, which females store in their spermathecae. These reproductive females, also known as queens in social hymenopterans, mate with one or several males and garner a lifetime supply of sperm. They never mate again, even though they may live and reproduce for decades [22]. Thus, low sperm quality and limited sperm production may have dramatic consequences for both male and female reproductive success.

**Figure 1.**
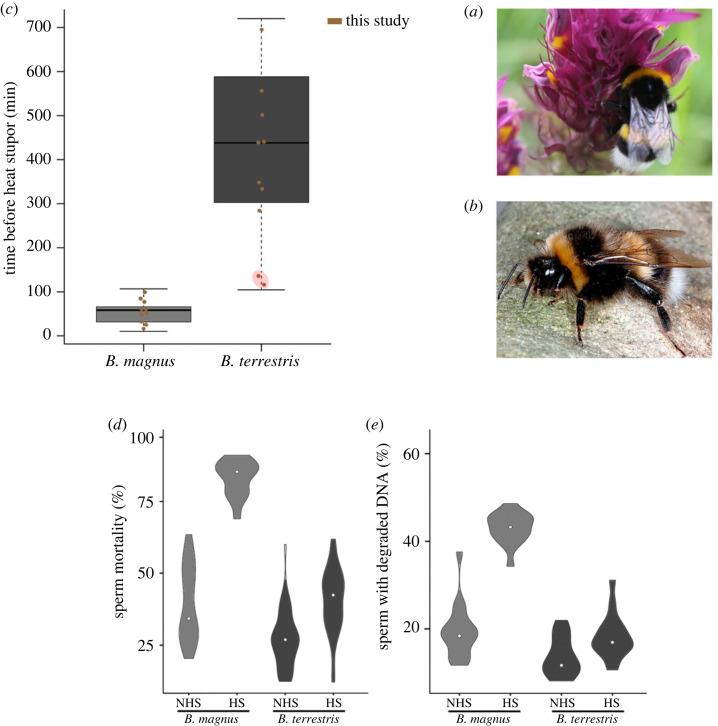
Heat tolerance in the bumblebees *Bombus terrestris* and *B. magnus* (modified from [15]). (*a*) *Bombus terrestris* (©M. Folschweiller). (*b*) *Bombus magnus* (©P. Rasmont). (*c*) Boxplots of time to heat stupor (THS) in *B. terrestris* and *B. magnus* (*n* = 30 for each species). The brown dots are the individual THS values. The two male *B. terrestris* with the lowest THS values (encircled in red) displayed protein expression profiles similar to those of *B. magnus* (figure 3). (*d*) Rate of sperm mortality and (*e*) relative amount of sperm with degraded DNA in *B. terrestris* and *B. magnus* subject to the control treatment (NHS) and the heat-stress treatment (HS). NHS = 12 h at 24°C, and HS = 40°C until heat stupor reached. The white dots are the median values for the treatment groups.

As keystone pollinators, bumblebees are essential to human populations. They ensure the reproduction of many flowering plants in regions with temperate and cold climates, and their global decline could cause major economic issues [23]. In most species, queens have a single mate. Therefore, the quality of the sperm they receive plays a determinant role in colony growth and fitness. Recently, heat stress was found to severely affect sperm quality in *B. magnus* by increasing DNA fragmentation and decreasing viability; however, no such effects were seen in *B. terrestris*, a sister species [15] (figure 1). The underlying causes of this difference remain unidentified.

Seminal fluid could help ensure that sperm survive thermal stress by positively affecting sperm functioning and providing a safe environment [24–26]. It contains high concentrations of sugars, a major energy source for sperm cells [27]. In addition, it contains proteins that influence a wide range of reproductive processes, including fertilization, sperm storage, immune responses, and, in some insect species, female physiology and behaviour [28].

Here, we investigated whether heat stress modified the seminal fluid proteomes of two bumblebee species. By characterizing interspecific differences in protein expression, we wished to mechanistically clarify how some species manage to keep their sperm alive in the face of extreme high temperatures.

## 2. Material and methods

### 2.1. Study species

*Bombus* (*B. sensu stricto*) *terrestris* (the buff-tailed bumblebee; figure 1*a*) is a Euro-Mediterranean species that has been spreading northward in Europe [29]. It is invasive in many parts of the world [30,31]. *Bombus* (*B. sensu stricto*) *magnus* (the northern white-tailed bumblebee; figure 1*b*) is a species found in regions with temperate or cold climates, and its populations are declining across most of its distribution range [32,33]. The two are sister species that occur sympatrically in some areas and that face the same ecoclimatic constraints. In 2021, we collected males of both species during their nuptial flights (patrolling) at two locations in Belgium (Kalmthout, 51° 22′ N, 04° 28′ E, elevation of 21 m; Maasmechelen, 50° 57′ N, 05° 41′ E, elevation of 39 m). Males take part in mating flights when they are 7–10 days old, the age at which sexual pheromone production peaks [34]. Consequently, all the males sampled were of comparable age. They were returned to the laboratory and maintained under controlled conditions: in constant darkness at 24°C with 50–60% relative humidity. They were fed a BIOGLUC sugar solution (Biobest NV, Westerlo, Belgium) until the experiment began 24 h later. As for species identification, all the samples were double-checked to ascertain their identification.

### 2.2. Heat-stress treatment

A heat-stress (HS) treatment was used to simulate the conditions that could occur during a heatwave, as described elsewhere [35]. Heatwaves involving temperatures of up to 40°C have occurred in more than 100 countries across the globe [36]. Consequently, male *B. magnus* (*n* = 20) and *B. terrestris* (*n* = 20) were individually exposed to a temperature of 40°C (relative humidity: 50–60%) under controlled conditions in an incubator (Herp Nursery II). We measured the time to heat stupor (THS), which served as a proxy for heat tolerance. THS was defined as the time between the bumblebee's placement in the incubator and the moment at which the insect fell on its back and was unable to right itself or express its normal reflexes (critical motor function) [37–39]. After reaching heat stupor, the bumblebees were removed from the incubator and allowed to recover. In the control treatment (NHS), males were kept at 24°C for 12 h, a period equivalent to the maximum THS seen in the HS treatment (figure 1*c*). All analyses related to the size were performed in R (version 4.0.3). Statistical analyses were conducted using linear models (lme4 package), with parameter significances reported as a t-distribution with estimated degrees of freedom.

### 2.3. Sperm extraction

Sperm were extracted from males (with similar size) in the HS and NHS groups 1 h after they had recovered from heat stupor. To stimulate the males, thumb pressure was gently applied several times to the entire length of sternite 1. Then, sternites 4 and 5 were pressed harder to fully expel the genitalia, including the sperm-filled endophallus. The base of the genitalia was pinched with forceps and gently pulled out of the abdominal cavity, along with the testes and accessory glands. Next, these organs were directly transferred to a Petri dish and doused with 70 µl of sodium phosphate buffer (PBS solution, pH 7.4). The endophallus was carefully dissected to release the sperm, which was collected and transferred into microtubes. This technique avoids extraction of proteins from the accessory glands that are not part of the contents transmitted to the female during copulation. To obtain the seminal fluid, the microtubes were centrifuged at 1100 r.p.m. and 4°C for 15 min; the resulting supernatant—the seminal fluid—was transferred to new microtubes. This approach minimized the seminal fluid from being contaminated with hemolymph or sperm [25]. The tubes were then immediately flash frozen in liquid nitrogen and stored at −80°C until protein extraction could occur. This procedure was repeated for each male which corresponds to one sample for the following proteomics analysis (*n* = 40).

### 2.4. Proteomics analysis of the seminal fluid

We characterized the males' HS responses using differential expression proteomics. Samples were sonicated for 3×10 s (amplitude = 40%; IKA U50 sonicator (Staufen, Germany)) in 70 µl of 6 M guanidinium hydrochloride and 25 mM Tris buffer (pH = 7.4). After centrifugation (15 min at 11 000 r.p.m.), the total amount of protein in a sample of the supernatant was determined using the Bradford protein assay; bovine gamma globulin was employed as a standard. Aliquots of protein (100 µg) were reduced with 0.21 M 1,4-dithioerythritol (5% v/v, 56°C, 20 min); alkylated with 0.5 M iodoacetamide (5% v/v, RT, 30 min); and precipitated with cold acetone (−20°C, overnight). The resulting protein pellets were solubilized using 50 mM ammonium bicarbonate containing 2 µg of trypsin (overnight at 37°C). Digestion was stopped with 0.5% formic acid (25% v/v, 0.1% final concentration). Peptide concentrations were measured using a Pierce Quantitative Colorimetric Peptide Assay (Thermo Fisher Scientific). We carried out mass spectrometry analyses using a concentration level of 0.8 µg μl^−1^ in 2% acetonitrile and 0.1% formic acid in water. Protein identification and quantification were performed using a label-free strategy and ultra-high-performance liquid chromatography–high resolution mass spectrometry (Eksigent NanoLC 425, AB SCIEX TripleTOF 6600+). Peptides (4 μg) were separated along a 15 cm C18 column (Triart C18, 3 µm, YMC) using a linear acetonitrile (I) gradient (5–35% v/v, 75 min) in water containing 0.1% (v/v) formic acid; the flow rate was 5 µl min^−1^. Mass spectra (MS) were acquired in high-resolution mode (>35 000) between 400 and 250 *m/z*; we used a 250 ms accumulation time. The instrument was operated in data-dependent acquisition (DDA) mode, and the MS/MS spectra were acquired between 100 and 1500 *m/z*. The precursor selection parameters were as follows: intensity threshold = 100 cps; maximum precursors per cycle = 90; accumulation time = 25 ms; and exclusion time after two spectra = 15 s. These parameters led to a 4 s duty cycle, which ensured that we obtained high-quality extracted ion chromatograms (XICs) to use for peptide quantification. ProteinPilot software (v. 5.0.1; ABSciex, USA) was employed to search databases for protein sequences. The query parameters included differential amino acid mass shifts for carbamidomethyl cysteine, biological modifications (i.e. oxidated methionine, *N*-terminal acetylation and glutamine deamidation) and missed trypsin cleavage sites. To construct the proteome, we performed protein identification runs by mapping identified peptides with different libraries among Hymenoptera in Proteome Discoverer. We downloaded a database of protein sequences from *Bombus*, *Apis* and *Osmia* from Uniprot website.

For the SWATH analyses, we defined 100 incremental steps using windows of variable values between 400 and 1250 *m/z*. The MS/MS working time for each window was 50 ms, resulting in a 5 s duty cycle. The ion chromatograms for the top six fragments of each peptide were obtained, and the areas under the curve (AUCs) were calculated. PeakView software (v. 2.1.0.11041; ABSciex, USA) was used in the SWATH processing of the proteins identified (FDR < 1%; determined by ProteinPilot 5.0.1). The retention time (RT) was recalibrated automatically using a PepCalMix standard (ABSciex, USA); RTs ranged from 20 to 85 min. Only proteins with 2 or more identifiable peptides were considered. A non-supervised principal components analysis (PCA) applying the Pareto principle was carried out to compare AUCs among groups (MarkerView™ 1.2.1, ABSciex, USA). Comparisons were only conducted when proteins displayed a fold change that was greater than 1.5 or less than 0.66 and for which the *p*-value was <0.05. All statistical analyses on the proteomics data were conducted in Protein Pilot 5.0.1 using simple two-group comparisons and a Benjamini–Hochberg correction. The functions of these proteins were explored by consulting the scientific literature as well as the Uniprot, KEGG and NCBI databases.

Gene ontology (GO) annotations for *Bombus* data [40,41] in the Hymenoptera Genome Database (available at hymenoptera.elsiklab.missouri.edu/hgd-go-annotation) were used for the GO and term enrichment analyses. For functional annotation of the sequences generated from the LC-MS/MS analyses, the GO terms were associated with all the genes based on the gene IDs of significantly upregulated and downregulated proteins. The GO terms for biological processes (BP) enriched were identified using the R package topGO v. 2.50.0 [42]. Terms displaying significant enrichment were identified ('weight01' algorithm, Fisher's exact test and alpha level of 0.05). We then verified the GO terms associated with BP for the genes in each cluster using g:Profiler [43] and including Bonferroni correction for significance threshold (*p*_adj_ < 0.05). We assessed gene involvement in specific biochemical and metabolic pathways using the Kyoto Encyclopedia of Genes and Genomes (KEGG), via the KEGG Automatic Annotation Server (KAAS) [44]. Separate lists of dysregulated proteins of the pairwise comparisons were loaded to investigate the biological pathways and the protein functions following heat exposure of bumblebee species.

## 3. Results

**Figure 2.**
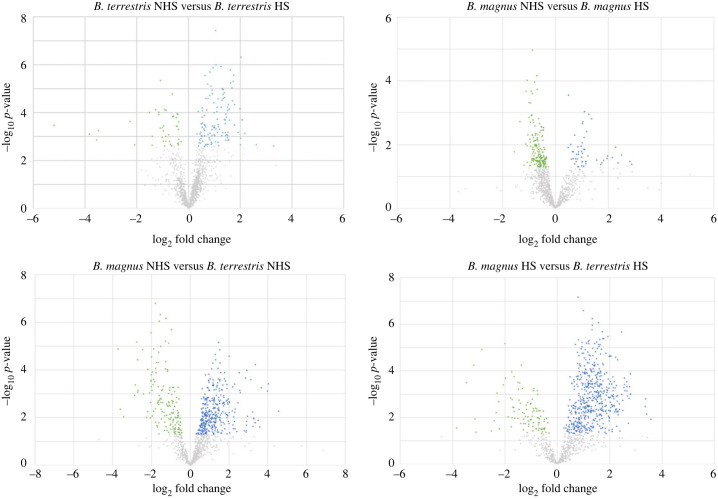
Volcano plots of seminal fluid protein expression in *B. terrestris* and *B. magnus*. Relative protein expression in the seminal fluid of male bumblebees experiencing the control treatment (NHS: 12 h at 24°C) versus the heat-stress treatment (HS: 40°C until heat stupor reached). The −log_10_(*p*-value) is plotted against the log_2_(fold change). A significant difference was defined as a protein displaying a ±1.5-fold change and *p* < 0.05 (prior to log transformation). The grey dots represent proteins that were not differentially expressed between groups (*p* > 0.05); the blue dots represent proteins whose expression significantly increased; and the green dots represent proteins whose expression significantly decreased.

**Figure 3.**
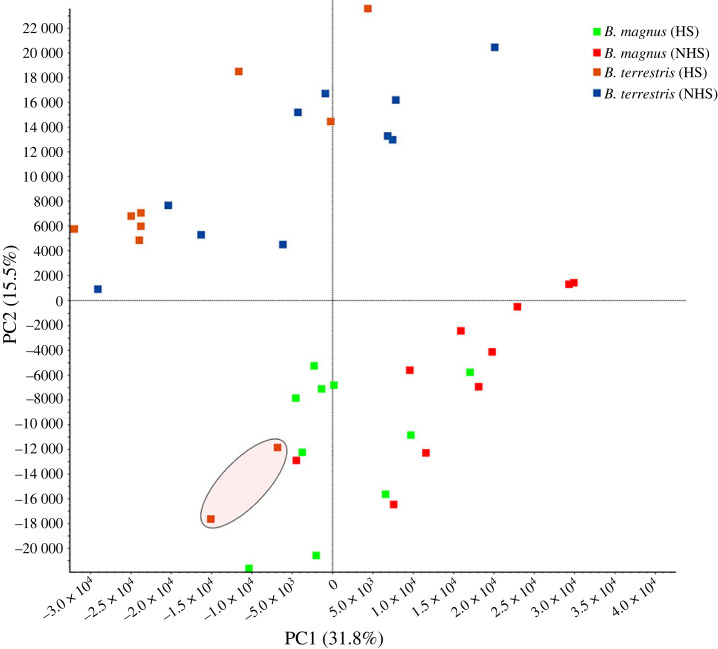
Principal component analysis. Expression of seminal fluid proteins in *B. terrestris* and *B. magnus*. Expression of seminal fluid proteins in male bumblebees experiencing the control treatment (NHS: 12 h at 24°C) versus the heat-stress treatment (HS: 40°C until heat stupor reached). Green: *B. magnus*—HS; red: *B. magnus*—NHS; orange: *B. terrestris*—HS; blue: *B. terrestris*—NHS. *n* = 10 for each species and treatment. The red circle encompasses the two male *B. terrestris* with the lowest THS values (figure 1*c*).

As already shown with the same methodology [45], we did not find a significant correlation between THS and the size of individuals (with dry weight as a proxy) for *B. terrestris* and *B. magnus* (respectively, *p* = 0.27 and *p* = 0.39). We identified and quantified 1121 proteins with at least two or more peptides in the seminal fluid of each of the two species (electronic supplementary material, file S2). The number of differentially expressed proteins (DEPs) varied more between species than within species between treatments (figure 2). Seminal fluid protein composition differed significantly between *B. terrestris* and *B. magnus* (perMANOVA, *p* < 0.001): there were 522 DEPs in the control treatment (167 with lower expression, 355 with higher expression) and 628 DEPs in the HS treatment (103 with lower expression, 525 with higher expression) (perMANOVA, *p* < 0.001). Within species, DEP numbers were much lower. Between treatments, there were 228 DEPs for *B. magnus* (175 with lower expression, 53 with higher expression) (perMANOVA, *p* < 0.001) and 168 DEPs for *B. terrestris* (43 with lower expression, 125 with higher expression) (perMANOVA, *p* < 0.01). Consequently, in *B. terrestris*, protein profiles differed by just 10% between the control and HS treatments (figure 2). A PCA of protein expression yielded similar results: there were two distinct clusters corresponding to the two species (figure 3). For *B. terrestris,* protein expression patterns were not distinct for the two treatment groups. By contrast, a clearer difference was seen for *B. magnus.* It is worth noting that two male *B. terrestris* from the HS group occurred in the *B. magnus* cluster, displaying the lowest THS values for their species (red circles in figure 1*c*). Males with THS values this small were previously shown to experience increased sperm mortality and DNA fragmentation (figure 1*d*,*e*).

The DEPs identified above were associated with the following functions involved in the HS response (table 1): protein folding (e.g. heat-shock proteins (HSPs), peptidylprolyl isomerase); cytoskeleton structure (e.g. myosin heavy-chain muscle, nesprin-1, spectrin, tubulin α-1 chain-like); signalling; antioxidant activity/defence (e.g. glutathione S-transferase, glutathione peroxidase); protein degradation (e.g. proteins involved in the ubiquitin proteasome system); lipid metabolism (e.g. neuroparsin-A isoform X1; short/medium-chain specific acyl-CoA dehydrogenase); mitochondrial respiratory chain complexes and DNA/RNA metabolism (e.g. Y-box factor homologue, DNA-directed RNA polymerase). These functional categories are linked to biological pathways responsible for preserving cellular homeostasis, like safeguarding and restoring the proteome, digesting and eliminating toxic residues, regulating the transcriptional machinery and DNA metabolism, and promoting cellular integrity [46]. The DEPs were expressed at much higher levels under conditions of heat stress in *B. terrestris* and, to a lesser extent, in *B. magnus.* Between the two species, the protein that displayed the greatest change was neuroparsin-A isoform X1, with a fold change of 0.997 for *B. terrestris*. Finally, compared to *B. magnus*, *B. terrestris* had much lower levels of proteins involved in mitochondrial respiratory chain complexes, and thus ROS production, in both treatments but had much higher levels of proteins involved in the capture of ROS. These proteins included the cytochrome b and c subunits, NADH dehydrogenase ubiquinone and NADH ubiquinone oxidoreductase.

**Table 1.** Proteins differentially expressed in *B. terrestris* and *B. magnus* in the control and heat-stress treatments and their functional categories. Only differentially expressed proteins (fold change < 0.66 or greater than 1.5 and *p* < 0.05) are described. Examples for each functional category are provided. Control treatment (NHS) = 12 h at 24°C; heat-stress treatment (HS) = 40°C until heat stupor reached.

	*B. terrestris* NHS and HS	*B. magnus* NHS	*B. magnus* HS
	example proteins	example proteins	example proteins
antioxidant activity/defence/signalling	superoxide dismutase [Cu-Zn]	peroxisomal N(1)-acetyl-spermine/spermidine	hydroxyacylglutathione hydrolase
	cytochrome c oxidase subunit 6b-3	oxidase	glutathione S-transferase
	cytochrome P450 6k1		cytochrome c oxidase subunit 6b-3
	lactoylglutathione lyase isoform X3		cytochrome b5-like isoform X2
	glutathione S-transferase		dehydrogenase/reductase SDR family member 4
cytoskeleton structure	myosin light chain alkali isoform X2	myosin heavy chain, non-muscle isoform X3	myosin heavy chain, muscle isoform X16
	tropomyosin-1	tubulin beta chain-like	tropomyosin-1, isoforms 9A/A/B isoform X14
	myosin-2 essential light chain isoform X3	tubulointerstitial nephritis antigen-like	myosin light chain alkali isoform X2
	microtubule-associated protein tau isoform X2		tubulin alpha; beta-1 chain-like profilin
	tubulin alpha-1 chain-like		
DNA/RNA metabolism	DNA-directed RNA polymerase II subunit RPB1	dnaJ homologue subfamily A member 1	serine-tRNA ligase, cytoplasmic
		ATP-dependent RNA helicase WM6	3'(2'),5'-bisphosphate nucleotidase 1
lipid metabolism	neuroparsin-A isoform X1	apolipophorins	acetyl-CoA acetyltransferase
	acyl-CoA-binding protein	enoyl-CoA delta isomerase 1	
	medium-chain specific acyl-CoA	acyl-CoA delta-9 desaturase	
	dehydrogenase	acetyl-CoA carboxylase isoform X7	
	isovaleryl-CoA dehydrogenase	3-hydroxyacyl-CoA dehydrogenase type-2-like	
	acetyl-CoA acetyltransferase		
protein degradation	ubiquitin-conjugating enzyme E2 L3 isof. X2	26S proteasome non-ATPase regulatory subunit 1, 2, 14	none
	ubiquitin carboxyl-terminal hydrolase	ubiquitin-like modifier-activating enzyme 1	
	kunitz-type serine protease inhibitor Bt-KTI	26S protease regulatory subunit 6A-B	
protein folding	heat shock 70 kDa, protein cognate 3, X3, 4	T-complex protein 1 subunit gamma, zeta, eta, beta	T-complex protein 1 subunit epsilon, delta, zeta
	28 kDa heat- and acid-stable phosphoprotein		
	heat shock protein 83		
	protein lethal(2)essential for life		

**Figure 4.**
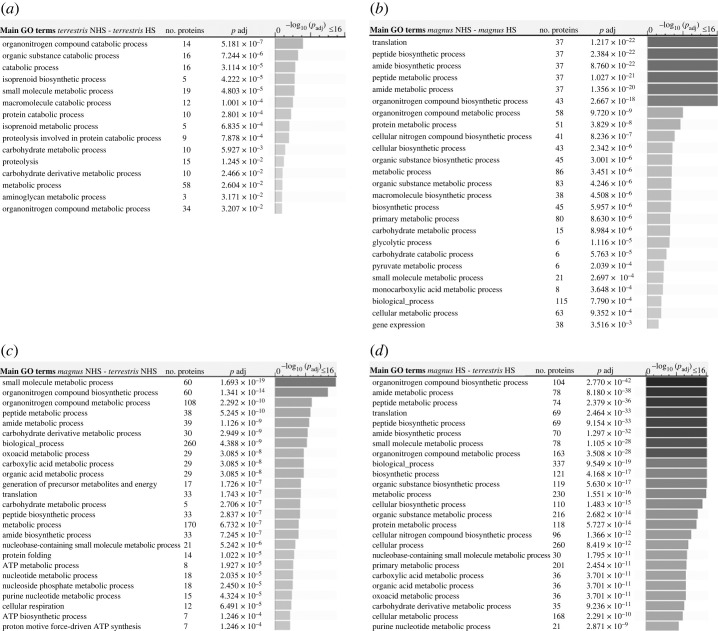
Functional distribution of the dysregulated proteins following heat exposure (40°C) according to the main impacted biological processes (BP) for the different pairwise comparison groups. Data for the first 25 gene ontology term enrichment are given. (*a*) *Bombus terrestris* NHS–*B. terrestris* HS; (*b*) *B. magnus* NHS–*B. magnus* HS; (*c*) *B. magnus* NHS–*B. terrestris* NHS; and (*d*) *B. magnus* HS–*B. terrestris* HS. In total, 15, 30, 64 and 91 GO terms were identified respectively for each pairwise comparison groups. Assignments were made with the R package topGO v. 2.50.0 and g:Profiler including Bonferroni correction for significance threshold (*p*_adj_ < 0.05). The complete lists of GO terms are available in electronic supplementary material, file S2. Control treatment (NHS) = 12 h at 24°C; heat-stress treatment (HS) = 40°C until heat stupor.

Functional annotation using GO terms was performed for the different pairwise comparison groups: (i) *B. terrestris* NHS–*B. terrestris* HS; (ii) *B. magnus* NHS–*B. magnus* HS; (iii) *B. magnus* NHS–*B. terrestris* NHS; and (iv) *B. magnus* HS–*B. terrestris* HS (figure 4). In total, we identified for each pairwise comparison groups 15, 30, 64 and 91 GO terms, respectively. For intraspecific comparisons the most affected BP for *B. terrestris* NHS–*B. terrestris* HS group (figure 4*a*) were catabolic process (organonitrogen compound, organic substance). For the group *B. magnus* NHS–*B. magnus* HS, more GO terms were identified, especially translation, peptide biosynthetic process, amide biosynthetic process, peptide metabolic process, amide metabolic process, organonitrogen compound biosynthetic process, organonitrogen compound metabolic process and protein metabolic process. For interspecific comparisons (*B. magnus* versus *B. terrestris*), both NHS and HS comparisons were characterized by many GO terms. For interspecific NHS comparison, small molecule metabolic process, organonitrogen compound biosynthetic process, organonitrogen compound metabolic process, peptide metabolic process, amide metabolic process, carbohydrate derivative metabolic process, biological process, oxoacid metabolic process, carboxylic acid metabolic process and organic acid metabolic process were found as BP. While for interspecific HS comparison, we found the following BP: organonitrogen compound biosynthetic process, amide metabolic process, peptide metabolic process, translation, peptide biosynthetic process, amide biosynthetic process, small molecule metabolic process, organonitrogen compound metabolic process, biological process, biosynthetic process, organic substance biosynthetic process, metabolic process, cellular biosynthetic process, organic substance metabolic process and protein metabolic process. All the BP were cited in order of significance (*p*_adj_). The complete lists of GO terms are available in electronic supplementary material, file S2.

Downregulated and upregulated proteins were significantly associated with 6 and 14 GO terms for BP, respectively, for *B. terrestris* NHS–*B. terrestris* HS; while they were significantly associated, respectively, with 28 and 2 GO terms for the comparison *B. magnus* NHS–*B. magnus* HS. For interspecific comparisons, we found 39 GO terms associated with downregulated proteins and 31 GO terms associated with upregulated proteins for *B. magnus* NHS–*B. terrestris* NHS comparison, while we found, respectively, 43 and 78 GO terms for *B. magnus* HS–*B. terrestris* HS comparison.

## 4. Discussion

Sperm cells are largely unable to maintain their own functionality, and it is likely that seminal fluid helps preserve sperm quality [24,25]. Our results are consistent with this hypothesis. We found that the protein composition of seminal fluid varied significantly between *B. terrestris* and *B. magnus,* two closely related bumblebee species whose sperm differ considerably in their vulnerability to heat stress (figure 1) [15].

Our data highlight the richness of seminal fluid proteins in bumblebees: 1121 proteins were identified in each of the two species, while using older mass spectrometry methods, Baer *et al*. [25] identified 57 proteins in the seminal fluid of honeybee drones. Our detailed analysis of seminal fluid protein expression in *B. terrestris* revealed the presence of numerous proteins that could potentially function to protect sperm against environmental stressors. In *B. magnus*, such proteins were not expressed at detectable levels in the control treatment. Even in the HS group, they were found at relatively low concentrations, compared to what was seen in *B. terrestris*.

Although we identified a similar number of seminal fluid proteins in both species, we found pronounced differences in their expression levels. In both the control and HS treatments, proteins with protective functions, such as HSPs, proteases and antioxidants, were differentially expressed to a greater degree in *B. terrestris* versus *B. magnus.* In the HS treatment, several proteins in different functional categories displayed higher expression in *B. magnus* (e.g. protein folding: T-complex protein 1 subunit epsilon, delta, zeta; antioxidant activity: hydroxyacylglutathione hydrolase, glutathione S-transferase, cytochrome c oxidase; lipid metabolism: acetyl-CoA acetyltransferase). However, the expression was much lower than seen in *B. terrestris*, even when compared with *B. terrestris* in the control treatment (table 1; electronic supplementary material, file S2). This finding suggests that, compared to male *B. magnus*, male *B. terrestris* may benefit from a better protection of their sperm cells that are more resistant to environmental stress. Support for this idea comes from previous research [15]: heat stress did not significantly affect sperm viability or DNA fragmentation in *B. terrestris* but did cause severe damage to sperm in more sensitive species as *B. magnus*. Furthermore, as shown in our GO term enrichment analyses, the heat exposure (NHS–HS) affected the regulation of fewer proteins associated with heat shock response in *B. terrestris* than in its sister species *B. magnus*. The relatively small changes in the protein composition of seminal fluid in *B. terrestris* suggest that this species is constitutively more capable of coping with thermal stress, thus maintaining sperm integrity and viability with more upregulated proteins associated with GO terms including carbohydrate metabolic process, generation of precursor metabolites and energy, protein folding and ATP biosynthetic process. The enriched GO terms related to proteostasis identified in *B. terrestris* were mostly trafficking and degradation (proteolysis), which are two key steps in autophagy [47], a cytoprotective process linked to cell viability [48].

There are a few key results to highlight. First, no matter the treatment, male *B. terrestris* displayed much higher levels of numerous proteins compared to *B. magnus*. These proteins were involved in maintaining cellular homeostasis, thermotolerance, antioxidant activity, lipid metabolism, sugar metabolism, protein folding (e.g. protein lethal(2)essential for life, HSPs), cytoskeleton structure (e.g. myosin, tubulin, profilin), defence (e.g. antimicrobial activity, antioxidant processes), protein degradation, lipid/sugar metabolism, DNA metabolism and RNA metabolism. These functional categories are known to contribute to the high thermotolerance of *Cataglyphis* desert ants [49,50], mites [51] and marine organisms [52]. Second, numerous proteins associated with mitochondrial respiratory chain complexes were differentially expressed between *B. terrestris* and *B. magnus*. Mitochondrial respiratory proteins are known to produce toxic ROS during heat stress [53]. Several of these proteins (e.g. cytochrome b561 isoform X2, NADH-ubiquinone oxidoreductase) displayed lower levels of expression in *B. terrestris*, meaning ROS production was constrained. More strikingly, proteins that capture and eliminate any ROS produced via mitochondrial respiration (e.g. cytochrome P450, cytochrome c oxidase, superoxide dismutase) were expressed at much higher levels in *B. terrestris* (control and HS treatments) than in *B. magnus*. Thus, seminal fluid proteins likely help balance ROS production and accumulation in sperm cells during and after heat stress [54]. Third, a wide range of HSPs were differentially expressed in the seminal fluid of male *B. terrestris* experiencing both treatments. These proteins included HSP 70 kDa, 28 kDa heat- and acid-stable phosphoprotein, HSP 83 and protein lethal(2)essential for life. By contrast, such was not the case in *B. magnus*. As a protein family, HSPs help maintain cell integrity during physiological stress. In insects, the basal levels of HSPs are correlated with environmental factors in a species-specific manner and are synthesized at different threshold temperatures (e.g. ants [55]; honeybees [56]; fruit flies [57]). Taken together, these differences in protein expression patterns may partly explain why high temperatures are much less of a threat to sperm viability and DNA integrity in *B. terrestris* than in *B. magnus* (figure 1*d*,*e*) [15].

Neuroparsin-A isoform X1 was one of the most overexpressed DEPs in *B. terrestris*: its expression was higher in the control and HS groups compared to *B. magnus* (fold change: 0.997). Neuroparsins are multifunctional neurohormones that inhibit the effects of juvenile hormones, stimulate fluid reabsorption in isolated recta and, more importantly, increase levels of lipids and trehalose in the hemolymph [58]. Since sugar is the main energy source for sperm [27], the higher concentrations of trehalose in the seminal fluid likely help maintain sperm viability under environmental stressors. As for lipids, they release substantial amounts of water when metabolized, which is thought to play a crucial role in both vertebrates and invertebrates facing heat and desiccation stress [59,60]. Unlike in *B. terrestris,* expression of neuroparsin-A did not differ between the two treatments in *B. magnus* and was much lower overall than in *B. terrestris* in general. Moreover, *B. terrestris* (NHS and HS) was characterized by the enrichment in GO terms associated with the metabolism of carbohydrates (e.g. trehalose) making sugar resources more available to spermatozoa, thereby playing a key role in sperm conservation. Future studies should explore this neurohormone's role in maintaining sperm quality in *B. terrestris*.

Little may be known about the molecular underpinnings of interspecific differences of seminal fluid content. While genetic and transcriptomic responses are well documented, much less attention has been paid to the expression of proteins, the actual molecular agents behind phenotypes [61]. Here, we showed that two bumblebees had very different seminal fluid proteomes and that their protein expression profiles fit with their ecological traits. *Bombus terrestris* is more broadly distributed and heat tolerant, while *B. magnus* is a species of concern that is heat sensitive. These differences could be mechanistically explained, at least in part, by differences in the vulnerability of their sperm to heat stress, a trait that could scale up to shape species resilience to global warming and extreme climatic events. However, it is important to note that our conclusions, while consistent with the literature and previous experiments on both species, are correlational. Further experiments based on functional tests to activate/inactivate some genes which could involve maintaining high sperm quality are needed to confirm our hypotheses if seminal proteome differences could cause the difference in heat-sensitivity of sperm between the bumblebee species.

To date, several risk factors have been identified that are contributing to the global decline of bumblebees, including changes in microbiota, diet, temperature and pesticide exposure [62–65]. However, many of these studies have focused on *B. terrestris*, a commercially available and invasive species [31] whose fitness seems to be only marginally affected by thermal stress [45,66]. The consequences of such stress could be far more dramatic in a sensitive species such as *B. magnus*. Thus, we risk obtaining biased results if we only use *B. terrestris* to extrapolate how bumblebees in general may cope with anthropogenic disturbances. Future studies must use a range of model species to explore the potential physiological and transgenerational impacts of environmental stressors on bumblebee reproduction [67]. Finally, bumblebee males live outside the nest and may be exposed to high temperatures during their courtship behaviour and nectar foraging. Further studies on sperm quality and the protein profile of the queen's spermathecal fluid (also important for fertilization success) after copulation are also needed to improve our overview of the effect of hyperthermal stress on queens, stored sperm and, ultimately, colony fitness.

## Data Availability

Protein data for *B. terrestris* and *B. magnus* are available in electronic supplementary material, files S1 and S2, describing the proteome regulation analyses (electronic supplementary material, file S1) and gene ontology enrichment analyses (electronic supplementary material, file S2) [68].

## Benefit-Sharing section

Benefits from this research accrue from the sharing of our data and results based on our methodology as described above.
